# Upregulation of CPT1A is essential for the tumor-promoting effect of adipocytes in colon cancer

**DOI:** 10.1038/s41419-020-02936-6

**Published:** 2020-09-10

**Authors:** Xiaopeng Xiong, Yang-An Wen, Rachelle Fairchild, Yekaterina Y. Zaytseva, Heidi L. Weiss, B. Mark Evers, Tianyan Gao

**Affiliations:** 1 Markey Cancer Center, University of Kentucky, Lexington, KY 40536-0679 USA; 2 Department of Toxicology and Cancer Biology, University of Kentucky, Lexington, KY 40536-0679 USA; 3 Department of Surgery, University of Kentucky, Lexington, KY 40536-0679 USA; 4 Department of Molecular and Cellular Biochemistry, University of Kentucky, Lexington, KY 40536-0679 USA

**Keywords:** Cancer metabolism, Lipid signalling

## Abstract

Colon tumors grow in an adipose tissue-enriched microenvironment. Locally advanced colon cancers often invade into surrounding adipose tissue with a direct contact with adipocytes. We have previously shown that adipocytes promote tumor growth by modulating cellular metabolism. Here we demonstrate that carnitine palmitoyltransferase I (CPT1A), a key enzyme controlling fatty acid oxidation (FAO), was upregulated in colon cancer cells upon exposure to adipocytes or fatty acids. In addition, CPT1A expression was increased in invasive tumor cells within the adipose tissue compared to tumors without direct contact with adipocytes. Silencing CPT1A abolished the protective effect provided by fatty acids against nutrient deprivation and reduced tumor organoid formation in 3D culture and the expression of genes associated with cancer stem cells downstream of Wnt/β-catenin. Mechanistically, CPT1A-dependent FAO promoted the acetylation and nuclear translocation of β-catenin. Furthermore, knockdown of CPT1A blocked the tumor-promoting effect of adipocytes in vivo and inhibited xenograft tumor initiation. Taken together, our findings identify CPT1A-depedent FAO as an essential metabolic pathway that enables the interaction between adipocytes and colon cancer cells.

## Introduction

Altered metabolism has been recognized as a common hallmark of cancer^1,2^. Emerging evidence indicates that the tumor microenvironment (TME) is essential in shaping the landscape of cancer metabolism^3^. A profile of the components in the TME revealed that adipocytes constitute a major cell type that is abundantly associated with tumor cells^4^. Metastatic colon cancer cells often encounter adipocytes as they first disseminate from their primary tumor site. Previous studies indicated that cancer-associated adipocytes promote tumor growth and progression by secreting growth factors and proinflammatory cytokines into the TME^5,6^. However, it is less understood how the direct interaction with adipocytes alters tumorigenic properties of cancer cells.

Recently, it has been shown that lipids produced in adipocytes can be transferred to cancer cells to promote tumor growth in ovarian cancer models, suggesting that local adipose tissues may have a direct role in supporting cancer cells^7^. Additionally, we reported that the uptake of fatty acids from adipocytes allows colon cancer cells to survive nutrient deprivation conditions by upregulating mitochondrial fatty acid oxidation (FAO)^8^. This transfer of fatty acids from adipocytes to cancer cells has been demonstrated in breast and melanoma cancer models as well^9,10^. Interestingly, the presence of cancer cells stimulates the release of fatty acids by promoting lipolysis in adipocytes, thus indicating a two-way communication between cancer cells and adipocytes in the TME^7,8^. Furthermore, treatment with fatty acids enhances the expression of genes associated with colon cancer stem cells (CSCs) and suppresses genes associated with intestinal epithelial cell differentiation^8^. This finding is consistent with the notion of CSC plasticity in that non-CSCs are capable of converting to CSCs given the right cue presented by the TME^11–13^. Taken together, these studies suggest that the close interaction between adipocytes and cancer cells plays an important role in regulating cancer metabolism.

Although the transfer of fatty acids from adipocytes to cancer cells has been confirmed in several studies^7–10^, the molecular mechanism underlying fatty acids-dependent metabolic regulation in cancer cells remains elusive. Here we further determined the role of CPT1A, a rate-limiting enzyme required for mitochondrial FAO, in mediating the tumor-promoting effect of adipocytes in colon cancer. Using primary colon cancer cells, 3D tumor organoids and in vivo xenograft models, we showed that uptake of fatty acids promotes the expression of CPT1A through the activation of PPARδ. Consequently, knockdown of CPT1A attenuated fatty acid utilization and eliminated the pro-survival advantage provided by adipocytes. In addition, we identified β-catenin acetylation as a novel mechanism connecting upregulation of FAO with increased Wnt/β-catenin signaling. Together, results from our study provided new mechanistic insights into the tumor-promoting effect of adipocytes in colon cancer.

## Materials and methods

### Cells and reagents

Patient-derived colon cancer PT130 cells were established as described previously^8,14^. Human colon cancer SW480 cells were purchased from ATCC. Both cell lines were maintained in DMEM supplemented with 10% fetal bovine serum (FBS, Sigma-Aldrich) and 1% penicillin–streptomycin. The cell lines were authenticated using short tandem repeat (STR) DNA profiling and tested negative for mycoplasma contamination (Genetica). The shRNA-targeting sequences for human CPT1A are as the following: 5′- GCCATGAAGCTCTTAGACAAA-3′ (C6), and 5′-CGATGTTACGACAGGTGGTTT-3′ (C7); and for mouse Cpt1a is: 5′-GCTATGGTGTTTCCTACATTA-3′. The following reagents were obtained from commercial sources as specified below: Acetyl-Coenzyme A Assay Kit, oleic acid (OA) (albumin complex), palmitic acid (PA), linoleic acid (LA) (albumin complex), octanoate, GW501516, GSK3787, and etomoxir (ETO) were from Sigma-Aldrich; BIODPY 493/503, N-2, and B-27 supplement were from Thermo Fisher Scientific. Bovine serum albumin (BSA)-conjugated palmitate was prepared according to the Seahorse protocol (Seahorse Bioscience). The concentrations of long chain fatty acids used in the cell treatment experiments (100–200 μM) are below the average plasma fatty acid concentrations found in healthy people^15^. For fatty acid treatment experiments, cells were cultured in low glucose media (5 mM, which is close to the normal blood glucose level) supplemented with 10% lipoprotein-deficient bovine serum (Alfa Aesar, J65182AMG). The octanoate concentration (3 mM) used for treating cells was based on the amount of octanoate needed to induce the expression genes related to “fatty acid metabolic process” as described previously^16^.

### Isolation of human mature adipocytes

Human omental or mesenteric fat tissues were collected from colon cancer patients undergoing surgery at the University of Kentucky Markey Cancer Center. The process for patients’ material collection was approved by the University of Kentucky’s Office for the Protection of Human Subjects. The isolation of adipocytes was carried out as described previously^8^. Equal amount of adipocytes were used as determined by the packed cell volume in all experiments (the density of purified adipocytes used is ~1–2 × 10^6^ cells/ml).

### Immunohistochemical (IHC) staining

Paraffin-embedded colon cancer patient specimens were obtained from the Biospecimen Procurement and Translational Pathology Shared Resource Facility of the Markey Cancer Center. The diagnosis and staging of each cancer case were confirmed by pathologist. IHC staining of paraffin-embedded tissue sections was performed as previously described^8,17^. The CPT1A antibody (#12252) was obtained from Cell Signaling. The stained sections were visualized using a Nikon Eclipse 80i upright microscope. To quantify the relative CPT1A expression levels, pixel intensity values were used to define the percentage of tumor cells with positive staining using the HALO image analysis platform (Indica Labs).

### Measurements of cellular acetyl-CoA (Ac-CoA) levels

The cellular levels of Ac-CoA were determined using the Acetyl-Coenzyme A Assay Kit (Sigma-Aldrich). Briefly, cells were cultured in low glucose media supplemented with 10% FBS. Total of 10^6^ cells were trypsinized, lysed in perchloric acid and neutralized using KHCO_3_. After centrifuging at 13,000 × *g* for 10 min, supernatants were collected and used for Ac-CoA measurements. The protein contents in the pellets were determined using the BCA Protein Assay Kit. The levels of Ac-CoA were presented as pmol/mg of protein.

### Seahorse extracellular flux analysis

The Seahorse XF96 Extracellular Flux Analyzer (Agilent) was used to measure the respiration activity of colon cancer cells as described previously^8,14,18^. The mitochondrial stress tests were performed according to manufacturer’s protocol. The relative levels of basal and maximal respiration were calculated based on OCR data obtained in the Mito stress tests. The FAO assays were performed as previously described with the following modifications^8^. Briefly, cells were seeded at the density of 3 × 10^4^ cells per well in a XF96 plate and subsequently incubated in substrate-limited medium (DMEM with 0.5 mM glucose, 1.0 mM glutamine, 0.5 mM carnitine, and 1% FBS) for ~16 h. Prior to the beginning of FAO measurements, the medium was switched into FAO assay medium (200 μM palmitate–BSA, 111 mM NaCl, 4.7 mM KCl, 2.0 mM MgSO_4_, 1.2 mM Na_2_HPO_4_, 2.5 mM glucose, 0.5 mM carnitine, and 5 mM HEPES). FCCP (3 μM), ETO (200 μM), and antimycin A (4 μM) were added subsequently at the indicated time. For measurements using OA–BSA as substrate, OA–BSA (100 μM) was added to the FAO assay medium replacing palmitate–BSA. All OCR measurements were normalized to total protein contents in each well. The basal, maximum, and reserved FAO were calculated based on OCR measurements upon the addition of mitochondrial inhibitors.

### Fatty acid degradation assay

Cells were seeded in 96-well plates at a density of 1 × 10^4^ cells per well and incubated with OA (200 μM) overnight. The cells were then switched to low glucose medium supplemented with 10% FBS and fixed in paraformaldehyde at indicated time points. To quantify the amount of lipids, the fixed cells were stained with BODIPY 493/503 (1 µg/ml) and the amount of BODIPY fluorescence was measured in cell lysates using a SpectraMax M5 Microplate Reader. For visualizing cellular lipid contents, cells were seeded onto coverslips in six-well plates, loaded with OA and allowed to unload for 24–48 h. The cells were then fixed and stained with BODIPY 493/503 and DAPI. The immunofluorescence images were obtained using a Nikon A1^+^ confocal microscope.

### Tumor organoid formation assay

Tumor organoids generated from Apc/Kras double mutant mice were cultured as described previously^8^. To silence Cpt1a expression, tumor organoids were dissociated into small cell clusters using TrypLE (Thermo) and incubated with sh-Cpt1a lentivirus in suspension for 6 h in a 37 °C incubator. Cells were subsequently embedded in 50% Matrigel in 3D growth medium (Advanced DMEM/F12 supplemented with 1 × Glutamax, 1 × N-2, 1 × B-27, 1 mM N-acetyl-l-cysteine, and 1% penicillin/streptomycin), and puromycin was added 2 days later to select for stable knockdown cells. To determine the tumor initiation capacity, organoids were dissociated to single cell suspensions using Accumax (Sigma-Aldrich). Total of 1000 cells per group were embedded in Matrigel as described above. The number of tumor organoids formed after 6 days were counted and analyzed. For gene expression analysis, tumor organoids were cultured in 3D Matrigel for 3 days and collected for RT-PCR^14^.

### Western blot analysis

Colon cancer cells or tumor organoids were collected and detergent-solubilized cell lysates were obtained as described previously^8,14^. The NE-PER Nuclear and Cytoplasmic Extraction Kit (Thermo) was used to separate cytoplasmic and nuclear fractions. Equal amounts of total cell lysates were resolved by SDS–PAGE and subjected to Western blot analysis. The following antibodies, including CPT1A, acetylated-lysine (pan-Lys, #9441) acetyl-histone H3 (Lys9) (#9649), acetyl-histone H3 (Lys27) (#8173), histone H3 (#14269), active-β-catenin (#8814), total β-catenin (#8480), were purchased from Cell Signaling; the acetyl-α-tubulin (T7451) and β-actin (A1978) antibodies were from Sigma-Aldrich; and the total α-tubulin (sc-5286) and Lamin A/C (sc-20681) antibodies were from Santa Cruz.

### Quantitative RT-PCR

Total mRNAs were isolated from cells using the RNeasy Mini Kit (Qiagen). Equal amounts of RNA were subjected to the reverse transcription PCR with the High Capacity cDNA Reverse Transcription kit (Thermo). The cDNAs obtained were subjected to RT-PCR reactions using the SYBR Green Master Mix (Thermo) and primers listed in Supplementary Table S1. To determine the expression of human and mouse CPT1A, gene-specific probes were purchased from Thermo and used in RT-PCR reactions using Taqman Gene Expression Master Mix (Thermo). All values were normalized to the level of β-actin.

### Xenograft tumorigenesis

All animal procedures were done using protocols approved by the University of Kentucky Animal Care and Use Committee. Six to eight week-old NOD.Cg-Prkdc^scid^ IL2rg^tm1Wjl^/SzJ (NSG, The Jackson Laboratory) mice were used. Both male and female mice of equal numbers were included in each group. Mice were housed in barrier rooms with 12-h light/dark cycle. For tumor growth assay, control and CPT1A knockdown SW480 cells in 5% Matrigel suspension (5 × 10^5^ cells in 100 µl) were mixed with 50 µl of freshly isolated human adipocytes (contain ~100,000 adipocytes) or PBS and inoculated subcutaneously. The tumor size was measured every week with a caliper, and the tumor volume was defined as (longest diameter) × (shortest diameter)^2^/2. At the end of experiments, tumors were harvested and subjected to mRNA and protein analysis. For tumor initiation assay, 100 or 1000 SW480 cells were mixed with Matrigel and adipocytes as described above and injected subcutaneously. The number of tumors formed was determined 3 months post injection.

### Statistical analysis

In experiments to assess rate of FAO, relative cell survival, mRNA expression, levels of Ac-CoA and colony formation were summarized using bar graphs and pairwise comparisons between different conditions were carried out using two-sample *t*-tests. For measuring the time course of fatty acid degradation, one-way or two-way analysis of variance models with two-way interaction terms for experimental factors such as treatment group, cell types and time were utilized. A linear mixed model was employed to compare slope of tumor volume growth curves over time between groups. The relative mRNA expression results represent average of three separate RT-PCR experiments with four replicates for each gene in each experiment. All other experiments were repeated three times and results shown represent the average of three experiments. Measurements of xenograft tumor growth were summarized at each time point of follow-up and analysis was performed using longitudinal models to account for repeatedly measured tumor volume over time within each mouse. The stem cell frequencies were calculated by extreme limiting dilution analysis (ELDA)^19^ using analytic tools available at http://bioinf.wehi.edu.au/ software/elda/.

## Results

### CTP1A expression is upregulated by fatty acids in colon cancer

**Fig. 1:**
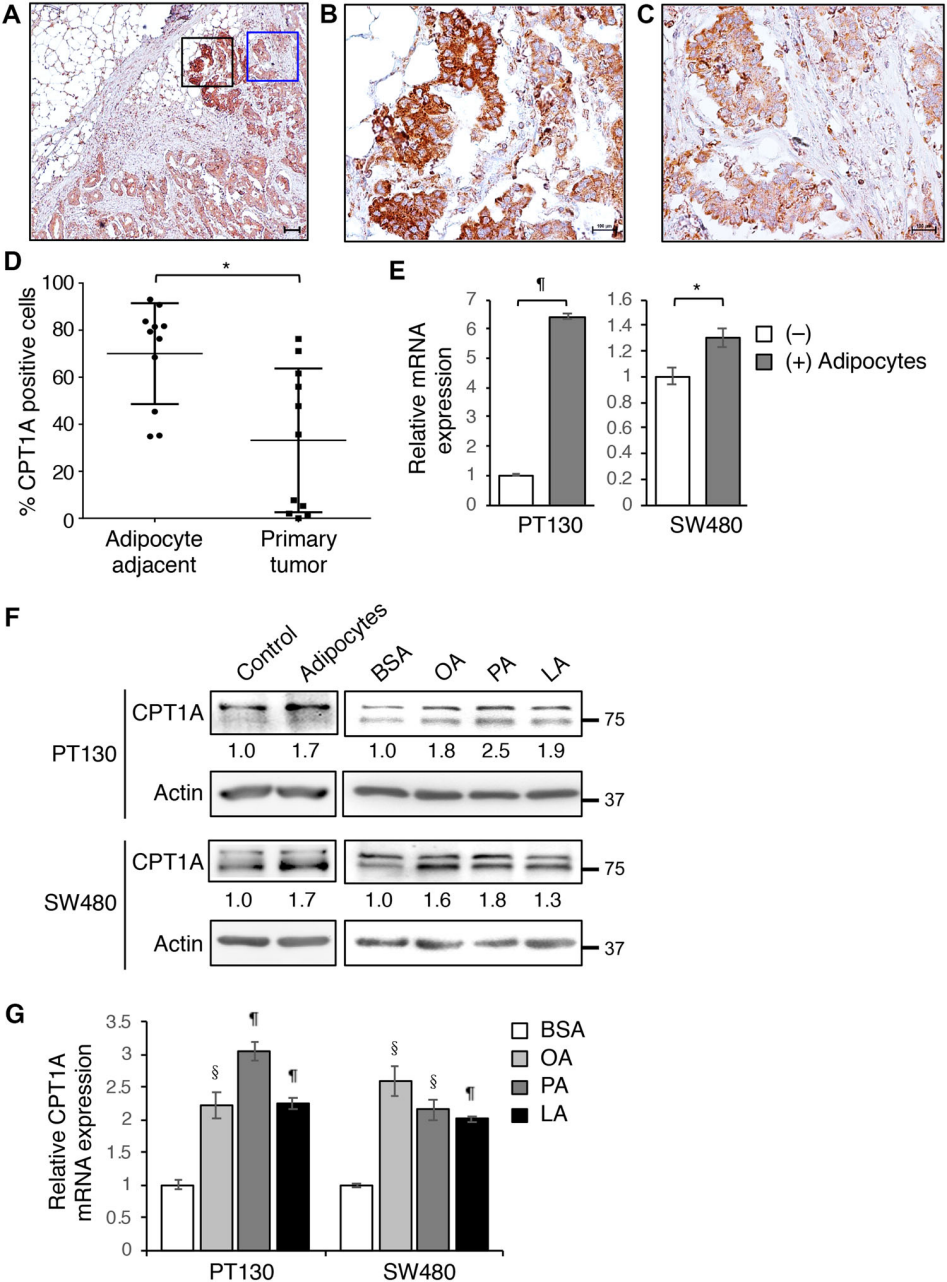
The expression of CTP1A is upregulated by fatty acids in colon cancer. **a**–**c** The expression of CPT1A protein was detected in a stage IV colon cancer patient tissues using IHC staining. The black-boxed and blue-boxed regions shown in **a** were enlarged and presented in **b** and **c**, respectively. Scale bar, 100 μm. **d** Quantitative analysis of CPT1A expression in specimens from 11 stage IV colon cancer patients. The percentage of invasive tumor cells in areas adjacent to adipocytes with positive CPT1A expression was higher than that of non-invasive primary tumors. Data represents mean ± SD (*n* = 11, *p < 0.01). **e** PT130 and SW480 cells were co-cultured with adipocytes isolated from colon cancer patients for 2 days. The levels of CPT1A mRNA expression were analyzed using RT-PCR. Data represents mean ± SD (*n* = 3, ^¶^*p* < 0.0001 and *p < 0.01). **f** Protein lysates prepared from cells co-cultured with adipocytes as described in **e** or treated with BSA, OA, PA, and LA (100 μM of each fatty acid species) for 24 h were analyzed for CPT1A protein expression using Western blotting. The relative levels of CPT1A were quantified by normalizing to β-actin and compared to control or BSA-treated cells. **g** PT130 and SW480 cells treated with BSA, OA, PA, or LA as described in **f** were analyzed for the expression of CPT1A mRNA using RT-PCR. Data represents mean ± SD (*n* = 3, ^§^*p* < 0.001 and ^¶^*p* < 0.0001).

We have shown previously that fatty acids released by adipocytes can be taken up by cancer cells to support cell survival by upregulating FAO^8^. Here we further investigated the mechanisms by which fatty acids induce this metabolic switch. To this end, we determined the expression of CPT1A in tumor specimens obtained from stage IV colon cancer patients. Interestingly, the expression of CPT1A was significantly increased in tumor cells that have invaded into the omental adipose tissue (adipocyte adjacent) compared to tumor cells without direct contact with adipocytes (primary tumors) (Fig. 1a–d and Supplementary Fig. S1). To examine the effect of fatty acids on CPT1A expression directly, we co-cultured PT130, a patient-derived colon cancer cell line, and SW480 cells with adipocytes. The CPT1A expression was markedly increased in the presence of adipocytes as measured by quantitative RT-PCR in colon cancer cells (Fig. 1e) and Western blotting analysis (Fig. 1f). Additionally, PT130 and SW480 cells were treated with OA, PA, or LA, three major fatty acid species found in human adipose tissues^20^, and the expression of CPT1A was consistently increased at both protein and mRNA levels (Fig. 1f, g). In subsequent experiments, OA or PA was used to examine CPT1A-dependent effects in colon cancer cells. Together, these results suggest that upregulation of CPT1A may provide a necessary mechanism for cancer cells to adapt a fatty acid-enriched microenvironment.

### Silencing CPT1A alters cellular metabolism in colon cancer cells

**Fig. 2:**
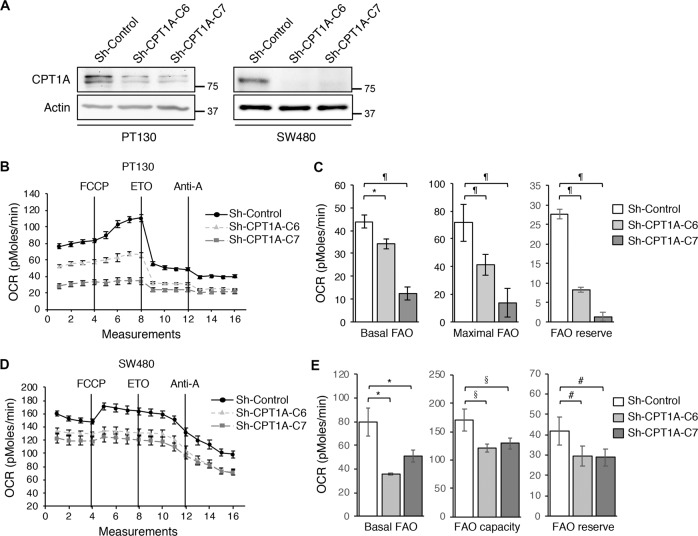
Knockdown of CPT1A inhibits fatty acid oxidation in colon cancer cells. **a** The expression of CPT1A protein was analyzed in stable control and CPT1A knockdown PT130 and SW480 cells using Western blotting. Two different shRNA targeting sequences (C6 and C7) were used to silence CPT1A in each cell line. β-actin was used as loading controls. **b** Representative OCR measurements obtained from the FAO tests performed in control (sh-Control) and CPT1A knockdown (sh-CPT1A-C6 and sh-CPT1A-C7) PT130 cells using the Seahorse XF96 Extracellular Flux analyzer. FCCP, ETO, and antimycin A (Anti-A) were added at the indicated points. **c** Experiments as shown in **b** were quantified and the relative levels of OCR associated with basal FAO, FAO capacity, and FAO reserve were calculated based on the measurements obtained from the addition of different compounds. Data represent the mean ± SD (*n* = 3, ^¶^*p* < 0.0001 and *p < 0.01). **d** Representative OCR measurements obtained from the FAO tests in sh-control, sh-CPT1A-C6, and sh-CPT1A-C7 SW480 cells using the Seahorse XF96 Extracellular Flux analyzer. **e** The relative levels of OCR associated with basal FAO, FAO capacity, and FAO reserve were quantified. Data represent the mean ± SD (*n* = 3, ^#^*p* < 0.05, *p < 0.01, and ^§^*p* < 0.001).

Given that CPT1A is the first rate-limiting enzyme in FAO, we next investigated the effect of silencing CPT1A on cellular metabolism. Stable CPT1A knockdown PT130 and SW480 cells were generated using two different lentiviral shRNAs (Fig. 2a). To determine the rate of FAO, the oxygen consumption rates (OCRs) were measured by using palmitate as the metabolic substrate in control and CPT1A knockdown cells with Seahorse XF96 Extracellular Flux Analyzer (Fig. 2b–e). We found that the OCRs associated with basal, maximal. and reserved FAO were significantly decreased in CPT1A knockdown PT130 and SW480 cells (Fig. 2c, e), confirming the role of CPT1A in controlling mitochondrial FAO. Similar results were obtained using OA as the metabolic substrate in Seahorse measurements to confirm that knockdown of CPT1A decreases FAO in colon cancer cells (Supplementary Fig. S2). In addition, since FAO and glycolysis are two functionally coupled metabolic processes that can be utilized by cancer cells, we determined if knockdown of CPT1A alters mitochondria-dependent glucose metabolism using Seahorse analysis (Supplementary Fig. S3). Interestingly, when using glucose as the metabolic substrate, results from Seahorse Mito Stress Tests showed that the OCRs associated with both basal and maximal mitochondrial respiration as well as ATP production were significantly increased in CTP1A knockdown cells (Supplementary Fig. S3A–D). Moreover, measurements from Seahorse Glycolysis Tests indicated that the extracellular acidification rate (ECAR) was increased in CTP1A knockdown cells (Supplementary Fig. S3E–H). These results are consistent with previous reports that ETO treatment can increase glucose uptake in normal and malignant cells^21,22^. Taken together, our results suggest that downregulation of CPT1A impairs FAO in colon cancer cells. However, this decreased FAO can be compensated by increased glucose metabolism. Indeed, we did not observe significant changes in the rate of cell proliferation in CTP1A knockdown cells when cultured in regular growth medium containing sufficient supplies of glucose (Supplementary Fig. S4).

### Knockdown CPT1A impairs fatty acid utilization and cell survival under nutrient deprivation conditions

**Fig. 3:**
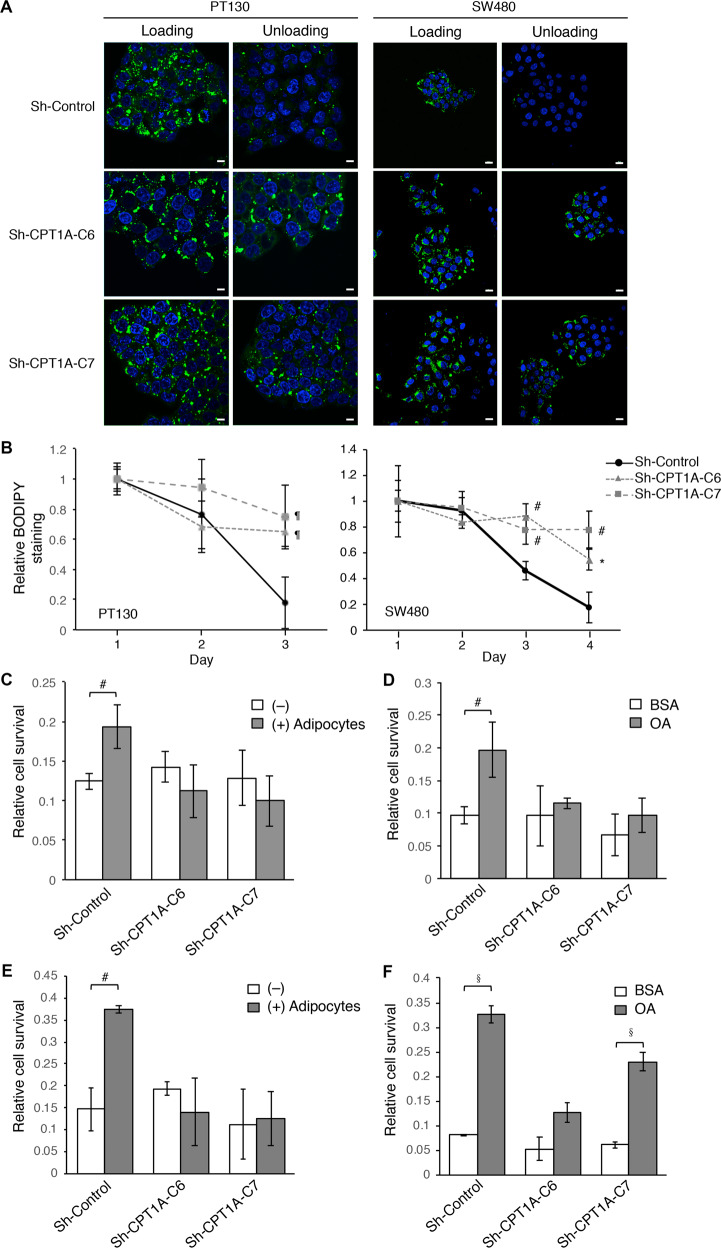
Knockdown of CPT1A impairs fatty acid utilization and cell survival under nutrient deprivation conditions. **a** Stable control (sh-Control) and CPT1A knockdown (sh-CPT1A-C6 and sh-CPT1A-C7) PT130 and SW480 cells were incubated with OA for 24 h (loading) and subsequently allowed to grow in regular growth medium for additional 24 or 48 h for PT130 and SW480 cells, respectively (unloading). Representative confocal images of cells stained with BODIPY 493/503 (green) and DAPI (blue). Scale bar, 100 μm. **b** Control and CPT1A knockdown PT130 and SW480 cells were incubated with OA for 24 h (day 1) and subsequently were stained with BODIPY 493/503 at indicated time points (days 2–4). The fluorescence intensity was measured using a fluorescence spectrophotometer as readout for relative lipid contents in cells. Data represent the mean ± SD (*n* = 3, ^#^*p* < 0.05, *p < 0.01, and ^¶^*p* < 0.0001). **c** and **d** Control and CPT1A knockdown PT130 cells were co-cultured with adipocytes **c** or pretreated with OA **d** for 24 h and subsequently cultured in EBSS for additional 48 h. The relative cell survival was measured using crystal violet staining. Data represent the mean ± SD (*n* = 3, ^#^*p* < 0.05). **e** and **f** Control and CPT1A knockdown SW480 cells were co-cultured with adipocytes **e** or pretreated with OA **f** for 24 h and subsequently cultured in EBSS for additional 48 h. The relative cell survival was determined. Data represent the mean ± SD (*n* = 3, ^#^*p* < 0.05 and ^§^*p* < 0.001).

To address the question if upregulation of CPT1A is necessary for cancer cells to utilize the exogenous fatty acids, control and CPT1A knockdown PT130 and SW480 cells were treated with OA for 24 h (the loading phase) and subsequently cultured in low glucose media for additional 24–48 h (the unloading phase) to allow the preferential utilization of fatty acids. Upon loading cells with OA, similar levels of lipid droplets accumulation were detected in control and CPT1A knockdown cells using BODIPY-493/505 staining (Fig. 3a). However, while BODIPY-staining of lipid droplets diminished in the unloading phase in control cells, significant levels of BODIPY-staining remained in CPT1A knockdown cells (Fig. 3a). Moreover, the relative lipid content was quantified by measuring BODIPY fluorescence intensity at different time points following OA treatment. The time course of fatty acids degradation was significantly delayed in CPT1A knockdown PT130 and SW480 cells as indicated by increased BODIPY retention compared to control cells (Fig. 3b).

Furthermore, we determined if silencing CPT1A disrupts the protective effect provided by fatty acid uptake. Control and CPT1A knockdown PT130 and SW480 cells were co-cultured with adipocytes or treated with OA for 24 h and subsequently cultured in EBSS buffer for additional 48 h (Fig. 3c–f). Our results showed that both co-culturing with adipocytes and OA treatment significantly increased the survival of control PT130 and SW480 cells under nutrient deprivation conditions. However, the pro-survival effect provided by adipocytes and fatty acids was largely abolished in CPT1A knockdown cells (Fig. 3c–f). Taken together, our results demonstrate that uptake of fatty acids renders colon cancer cells resistant to nutrient deprivation and this acquired survival advantage relies on CPT1A-mediated FAO.

### Downregulation of Cpt1a reduce cancer stem cell properties in 3D tumor organoids

**Fig. 4:**
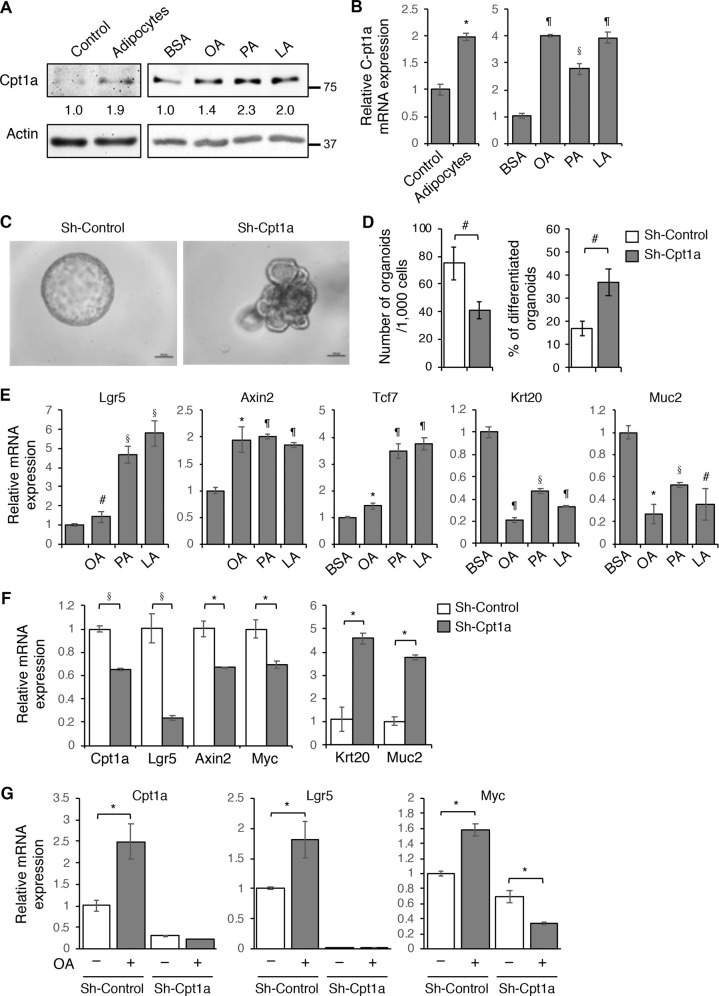
CPT1A-dependent fatty acid oxidation promotes Wnt signaling and cancer stem cell properties. **a** Tumor organoids derived from Apc/Kras double mutant mice were embedded with adipocytes in 3D Matrigel or treated with BSA, OA, PA, and LA (100 μM each) for 2 days. The levels of Cpt1a expression were analyzed using Western blotting. The relative levels of CPT1A were quantified by normalizing to β-actin and compared to control or BSA-treated cells. **b** Tumor organoids treated as described in **a** were analyzed for the expression of *Cpt1a* mRNA using RT-PCR. Data represents mean ± SD (*n* = 3, *p < 0.01, ^§^*p* < 0.001, and ^¶^*p* < 0.0001). **c** The expression of Cpt1a was silenced in Apc/Kras mutant tumor organoids using lentiviral shRNA. Single cell suspensions of control and Cpt1a knockdown cells were seeded in 3D Matrigel. Representative images of control and Cpt1a knockdown tumor organoids are shown after 6 days in culture. Scale bar, 100 μm. **d** The number of tumor organoids formed and the percentage of organoids showed branching phenotype were quantified (total 1000 cells were seeded per group). Data represent the mean ± SD (*n* = 3, ^#^*p* < 0.05). **e** Tumor organoids treated with BSA, OA, PA, and LA (100 μM each) were analyzed for the expression of Wnt β-catenin target genes (including Lgr5, Axin2, and Tcf7) and genes associated with intestinal epithelial cell differentiation (including Krt20 and Muc2) using RT-PCR. Data represents mean ± SD (*n* = 3, ^#^*p* < 0.05, *p < 0.01, ^§^*p* < 0.001, and ^¶^*p* < 0.0001). **f** Control and Cpt1a knockdown organoids were subseeded and grown in 3D Matrigel for 3 days. The expression of Cpt1a, Wnt/β-catenin target genes (including Lgr5, Axin2, and Myc), and genes associated with intestinal epithelial cell differentiation (including Krt20 and Muc2) was determined using RT-PCR. Data represent the mean ± SD (*n* = 3, *p < 0.01 and ^§^*p* < 0.001). **g** Control and Cpt1a knockdown organoids were treated with OA for 2 days. The mRNA expression of Cpt1a as well as target genes of Wnt/β-catenin (including Lgr5 and Myc) was determined using RT-PCR. Data represent the mean ± SD (*n* = 3, *p < 0.01).

We have previously shown that the presence of adipocytes increases the expression of genes associated with cancer stem cells in intestinal tumor organoids derived from Apc/Kras double mutant mice^8^. Here we investigated if upregulation of CPT1A is required to mediate the effect of adipocytes on promoting cancer stem cell functions. First, the Apc/Kras tumor organoids were embedded with adipocytes in 3D Matrigel or treated with different fatty acids and the expression of Cpt1A was monitored using Western blot and RT-PCR analysis. Consistent with results shown in Fig. 1, the presence of adipocytes or fatty acids increased Cpt1a expression at both protein and mRNA levels (Fig. 4a, b). Next, we silenced the expression of Cpt1a using lentiviral shRNA in Apc/Kras organoids. Interestingly, while control tumor cells formed spherical organoids in 3D, the Cpt1a knockdown organoids showed branched phenotype suggesting potential differentiation (Fig. 4c). Moreover, single cell suspensions of control and Cpt1a knockdown cells were seeded in 3D Matrigel and the numbers of colonies formed were determined after 6 days. We found that the ability of Cpt1a knockdown cells to form colonies in 3D was significantly decreased whereas the percentage of organoids with the branched phenotype was increased (Fig. 4d). Similar reduction in colony formation and increase in differentiation were observed in Apc/Kras organoids treated with ETO, an inhibitor of CPT1A (Supplementary Fig. S5A and B). In addition, since the 3D growth media are enriched in fatty acids, silencing Cpt1a also reduced the rate of cell proliferation in tumor organoids (Supplementary Fig. S5C). However, the reduced colony formation was not due to decreased cell survival as knockdown of Cpt1a had no effect on cell viability (Supplementary Fig. S5D).

Consistent with the notion that Wnt signaling activation is required to promote cancer stem cell properties^23,24^, we found that treating tumor organoids with three different fatty acids significantly increased expression of Wnt target genes and decreased genes associated with intestinal epithelial cell differentiation (Fig. 4e). Moreover, knockdown of Cpt1a largely reduced the expression of genes associated with CSCs downstream of Wnt/β-catenin and increased the expression of differentiation markers (Fig. 4f). Furthermore, while fatty acid treatment significantly increased the expression of Wnt target genes, silencing Cpt1a expression abolished the effect of fatty acids (Fig. 4g). Collectively, our results suggest that fatty acid treatment enhances Wnt signaling in tumor organoids using a CPT1A-dependent mechanism.

### Fatty acids increase CPT1A expression and Wnt signaling through PPARδ in colon cancer

**Fig. 5:**
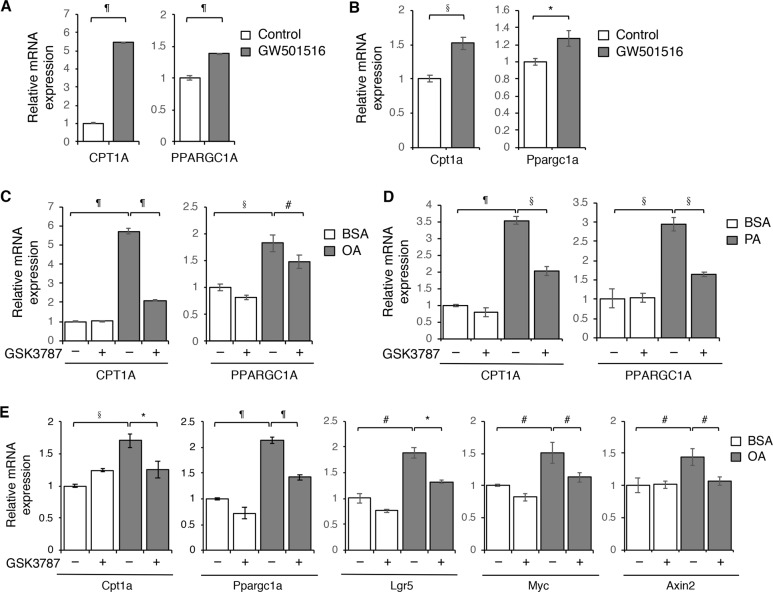
Fatty acids activate PPARδ to promote CPT1A expression and Wnt signaling in colon cancer cells. **a** PT130 cells were treated with PPARδ agonist GW501516 (1 μM) for 24 h. The expression of CPT1A and PPARGC1A was determined using RT-PCR. Data represent the mean ± SD (*n* = 3, ^¶^*p* < 0.0001). **b** Apc/Kras tumor organoids were treated with PPARδ agonist GW501516 (1 μM) for 48 h. The expression of Cpt1a and Ppargc1a was determined using RT-PCR. Data represent the mean ± SD (*n* = 3, *p < 0.01 and ^§^*p* < 0.001). **c** PT130 cells were treated with OA alone or in combination with PPARδ antagonist GSK3787 (1 μM) for 24 h. The expression of CPT1A was determined using RT-PCR. Data represent the mean ± SD (*n* = 3, ^#^*p* < 0.05,^§^*p* < 0.001, and ^¶^*p* < 0.0001). **d** PT130 cells were treated with PA alone or in combination with PPARδ antagonist GSK3787 (1 μM) for 24 h. The expression of CPT1A and PPARGC1A was determined using RT-PCR. Data represent the mean ± SD (*n* = 3, §*p* < 0.001 and ^¶^*p* < 0.0001). **e** Apc/Kras tumor organoids were treated with OA alone or in combination with PPARδ antagonist GSK3787 (1 μM) for 48 h. The expression of Cpt1a, Ppargc1a, and Wnt/β-catenin target genes (including Lgr5, Myc, and Axin 2) was determined using RT-PCR. Data represent the mean ± SD (*n* = 3, ^#^*p* < 0.05, *p < 0.01, ^§^*p* < 0.001, and ^¶^*p* < 0.0001).

To determine the molecular mechanism by which fatty acids and adipocytes promote CPT1A expression, we investigated the functional contribution of peroxisome proliferator-activated receptor δ (PPARδ). PPARδ is a lipid sensing nuclear receptor that can be activated by long-chain fatty acids to regulate cellular metabolism^25^. Previous studies have shown that PPARδ play a critical role in controlling FAO in muscle or adipocytes tissues and high-fat diet enhance tumor initiation potential of intestinal organoids in by activating PPARδ^26,27^. To confirm the involvement of PPARδ in regulating CPT1A, we showed that treating PT130 cells or Apc/Kras tumor organoids with PPARδ agonist GW501516 directly stimulated the expression of CPT1A and PPARGC1A (a known PPARδ target gene) (Fig. 5a, b). In addition, fatty acid-induced upregulation of CPT1A was effectively blocked by pretreating cells with PPARδ antagonist GSK3787 (Fig. 5c, d). Importantly, inhibition of fatty acid-induced PPARδ activation decreased the expression of Wnt-targeting genes (Fig. 5e) in tumor organoids. Collectively, our data indicate that fatty acids stimulate CPT1A expression to enhance FAO and Wnt signaling mainly through PPARδ activation in colon cancer cells.

### Downregulation of CPT1A decreases β-catenin acetylation and activation

**Fig. 6:**
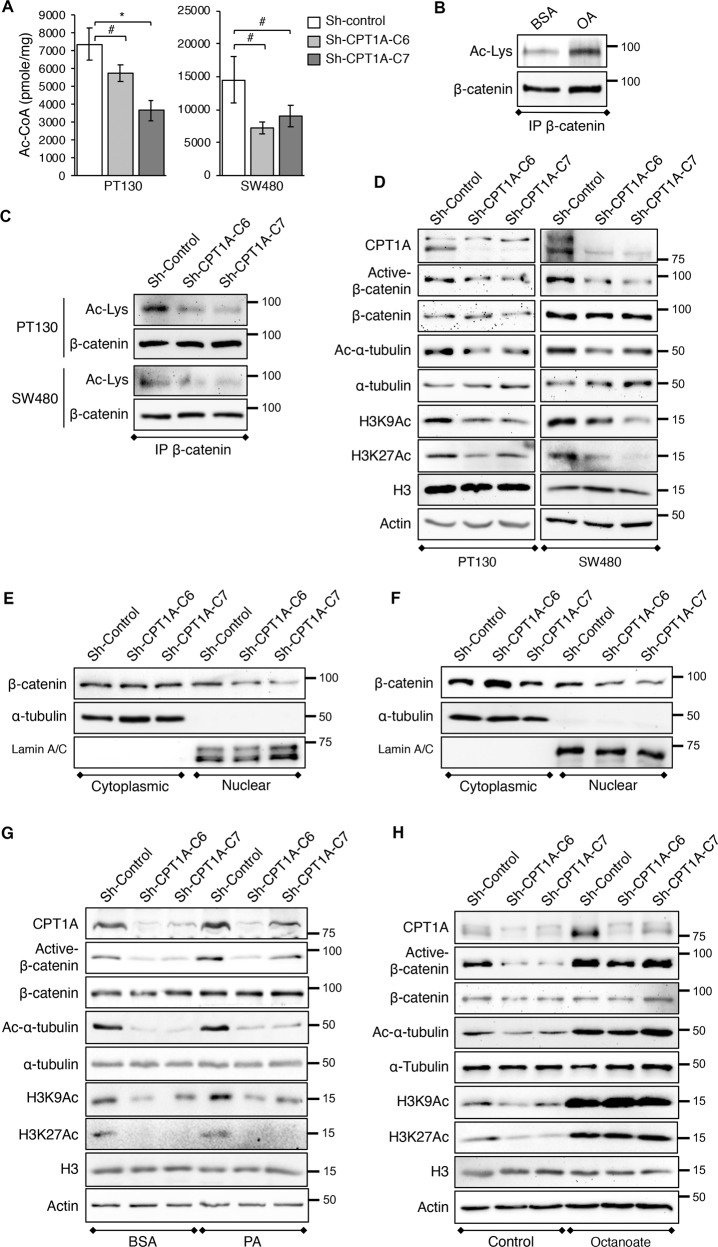
Downregulation of CPT1A decreases cellular levels of acetyl-CoA and protein acetylation. **a** Control and CPT1A knockdown PT130 and SW480 cells were cultured in low glucose media supplemented with 10% FBS. The levels of acetyl-CoA in control and CPT1A knockdown PT130 and SW480 cells were determined using the Acetyl-CoA Assay kit. Data represent mean ± SD (*n* = 3, ^#^*p* < 0.05 and *p < 0.01). **b** PT130 cells were treated with BSA or OA (100 μM) for 24 h. Cell lysates were immunoprecipitated using the anti-β-catenin antibody and analyzed for the levels of β-catenin acetylation using the acetylated-lysine antibody (Ac-Lys). The blot was stripped and reprobed for total β-catenin in the immunoprecipitates. **c** Cell lysates from control and CPT1A knockdown PT130 or SW480 cells were immunoprecipitated and analyzed for the levels of β-catenin acetylation using the acetylated-lysine antibody (Ac-Lys). The blot was stripped and reprobed for total β-catenin in the immunoprecipitates. **d** Cell lysates from control and CPT1A knockdown PT130 or SW480 cells were analyzed for the levels of active-β-catenin as well as acetyl-α-tubulin (Ac-α-tubulin), acetyl-histone at K9 and K27 residues (H3K9Ac and H3K27Ac) using Western blotting. Total β-catenin, α-tubulin, histone H3, and β-actin were used as loading controls. **e** and **f** Control and CPT1A knockdown PT130 **e** and SW480 **f** cells were fractionated into cytoplasmic and nuclear fractions. The amount of β-catenin in each fraction was determined using the anti-β-catenin antibody. α-Tubulin and lamin A/C were used as controls for the cytoplasmic and nuclear fractions, respectively. **g** Control and CPT1A knockdown PT130 cells were treated with BSA or PA (100 μM) for 24 h and cell lysates were analyzed for the levels of active-β-catenin as well as Ac-α-tubulin, H3K9Ac, and H3K27Ac using Western blotting. **h** Control and CPT1A knockdown PT130 cells were cultured under control condition or treated with octanoate (3 mM) for 24 h and cell lysates were analyzed for the levels of active-β-catenin as well as Ac-α-tubulin, H3K9Ac, and H3K27Ac using Western blotting.

We next determined the mechanism underlying fatty acid-induced activation of Wnt signaling. The FAO has been identified as a major carbon source for the production of cellular Ac-CoA, a central metabolite that regulates protein acetylation^16,28^. Here we examined whether CPT1A downregulation alters cellular Ac-CoA levels. As shown in Fig. 6a, knockdown of CPT1A significantly reduced total Ac-CoA levels in both PT130 and SW480 cells. Given that fatty acid-derived Ac-CoA can be used as substrate for protein acetylation and acetylation of β-catenin enhances Wnt signaling by promoting β-catenin nuclear localization^29–31^, we investigated whether the reduced acetyl-CoA production affects β-catenin activity. To this end, endogenous β-catenin proteins were immunoprecipitated from PT130 cells treated with BSA or OA and the level of β-catenin acetylation was determined using an acetylated-lysine antibody. Indeed, treatment with OA increased β-catenin acetylation (Fig. 6b). Moreover, knockdown of CPT1A markedly reduced levels of β-catenin acetylation in PT130 and SW480 cells suggesting CPT1A-dependent FAO controls β-catenin acetylation (Fig. 6c). Consistent with the notion that acetylation of β-catenin increases its activity, results from Western blot analysis showed that the levels of active β-catenin (non-phosphorylated form) were decreased in CPT1A knockdown PT130 and SW480 cells (Fig. 6d). As controls, we analyzed the acetylation status of α-tubulin, a cytoplasmic substrate, and histone H3, a nuclear substrate, at lysine 9 (H3K9) and lysine 27 (H3K27) residues. Consistently, the levels of Ac-α-tubulin as well as H3K9Ac and H3K27Ac were reduced in CPT1A knockdown cells confirming that FAO is important for regulating a broad range of protein acetylation events (Fig. 6d). Similar decrease in the levels of active β-catenin and acetylated α-tubulin, H3K9 and H3K27 was observed in Cpt1a knockdown tumor organoids (Supplementary Fig. S6A). To further determine the functional effect of decreased β-catenin acetylation, control and CPT1A knockdown PT130 and SW480 cells were fractionated into cytoplasmic and nuclear fractions. The amount of β-catenin in the nuclear factions was decreased in CPT1A knockdown PT130 and SW480 cells confirming that acetylation of β-catenin promotes its nuclear localization (Fig. 6e, f).

Furthermore, control and CPT1A knockdown PT130 cells treated with PA were subjected to Western blot analysis to determine the functional contribution of CPT1A-dependent FAO in regulating protein acetylation. Results showed that PA treatment increased levels of active β-catenin as well as acetylated α-tubulin, H3K9, and H3K27 in control cells whereas silencing CPT1A blocked the effect of fatty acids on β-catenin activation and protein acetylation (Fig. 6g). Similar results were obtained in control and CPT1A knockdown cells treated with OA (Supplementary Fig. S6B). As a control, we treated cells with octanoate, a medium-chain fatty acid that can enter mitochondria and undergo FAO independent of CPT1A^16^. Interestingly, octanoate treatment markedly increased the levels of acetylated α-tubulin, H3K9, and H3K27 as well as active β-catenin in both control and CPT1A knockdown cells (Fig. 6h). Consistent with a dose-dependent effect of octanoate on modulating protein acetylation^16^, we showed that treating cells with a lower concentration of octanoate partially rescued the acetylation defect observed in CPT1A knockdown cells (Supplementary Fig. S6C). Taken together, our results suggest the acetyl-CoA derived from long-chain FAO can be used as the substrate for protein acetylation in a CPT1A-dependent manner. Because free fatty acids derived from adipocytes are in the form of long-chain fatty acids, upregulation of CPT1A allows the cancer cells to produce increasing levels of Ac-CoA via mitochondrial FAO to promote β-catenin activation and Wnt signaling.

### Knockdown of CPT1A inhibits xenograft tumor growth and tumor initiation in vivo

**Fig. 7:**
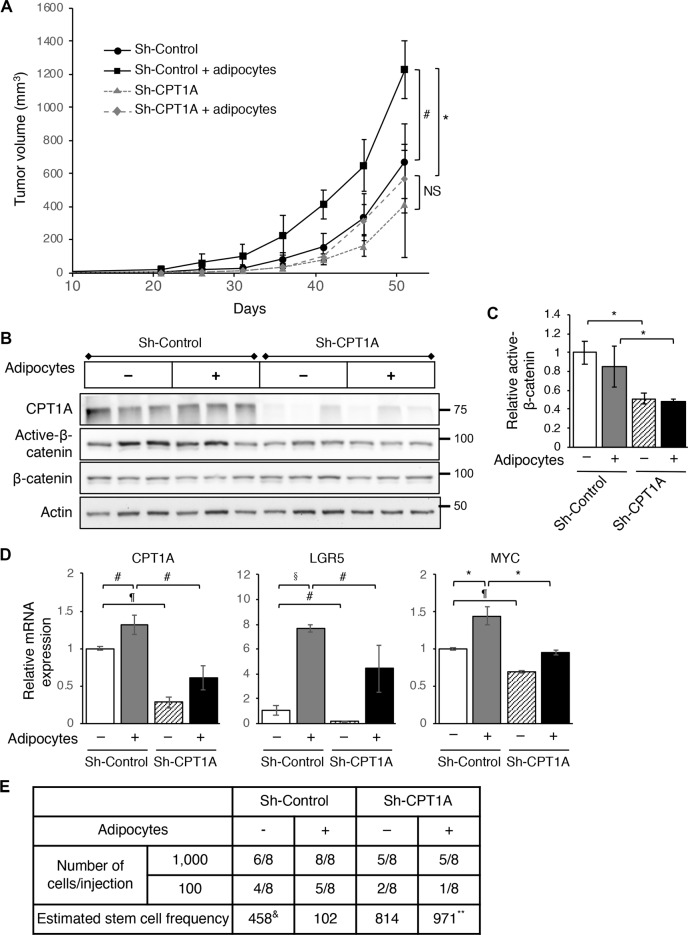
CPT1A is required to mediate the tumor promoting effect of adipocytes in vivo. **a** Control and CPT1A knockdown SW480 cells were mixed with Matrigel alone or in combination with adipocytes and injected subcutaneously into NSG mice. The size of the tumors was measured every 5 days for 51 days. Data represent the mean ± SEM (*n* = 6, for sh-Control and sh-CPT1A group; and *n* = 7 for sh-Control + adipocytes and sh-CPT1A + adipocytes group, ^#^*p* < 0.05 and *p < 0.01; NS = not significant). **b** Tumor tissues from three mice of each group were analyzed for the levels of CPT1A, active-β-catenin, and β-catenin using Western blotting. **c** Quantitative analysis of relative active-β-catenin levels in xenograft tumors from four different groups of mice. The levels of active-β-catenin were normalized to total β-catenin in each sample. Data represent the mean ± SD (*n* = 3, *p < 0.01). **d** Tumor tissues from three mice of each group were analyzed for the expression of CPT1A, LGR5, and MYC using RT-PCR. Data represent the mean ± SD (*n* = 3, ^#^*p* < 0.05, *p < 0.01, §*p* < 0.001, and ^¶^*p* < 0.0001). **e** Tumor initiation experiments were performed using control and CPT1A knockdown SW480 cells. Cells were mixed with Matrigel alone or in combination with adipocytes and injected into NSG mice at 100 or 1000 cells per site and total eight injections were used for each cell group. The number of tumors formed was determined 3 months post inoculation. The stem cell frequency was calculated using extreme limiting dilution analysis (ELDA) (&*p* = 0.01, comparing sh-control group with sh-control + adipocytes group; and **p = 0.0003, comparing sh-control + adipocytes group with sh-CPT1A + adipocytes group).

To examine the effect of silencing CPT1A on tumor growth in vivo, we subcutaneously injected control and CPT1A knockdown SW480 cells mixed with Matrigel alone or in combination with human adipocytes into NSG mice and monitor the tumorigenesis process. Consistently with the tumor promoting effect of adipocytes, the rate of tumor growth was largely increased when the control SW480 cells were co-injected with adipocytes (Fig. 7a). Although knockdown of CPT1A did not significantly decrease the tumor growth rate basally compared to the control group, CPT1A-loss blocked the tumor promoting effect of adipocytes in vivo (Fig. 7a). Analysis of tumor tissues collected from four groups of mice revealed that knockdown of CPT1A decreased the amount of active β-catenin, although we did not observe the adipocyte-induced activation of β-catenin in tumors derived from control cells (Fig. 7b, c). It is likely that the effect of adipocytes on promoting protein acetylation gradually diminished as adipocytes did not expect to survive the entire tumorigenesis process. Nevertheless, results from RT-PCR analysis showed that the expression of CPT1A and Wnt target genes (including LGR5 and MYC) was elevated in control SW480 cells that co-injected with adipocytes and knockdown of CPT1A largely abolished this effect (Fig. 7d).

Furthermore, we performed tumor initiation experiments by injecting control and CPT1A knockdown SW480 cells mixed with Matrigel alone or in combination with adipocytes into NSG mice at 100 and 1000 cells per site. The number of tumors formed was determined after 3 months. Based on an ELDA, co-injection of adipocytes significantly increased the stem cell frequency in control cells (from 1 in 458 basally to 1 in 102 in the presence of adipocytes) but not in CPT1A knockdown cells (Fig. 7e). Taken together, our results suggest that CPT1A is required for adipocytes to enhance the tumor initiation potential in vivo.

In summary, results from this study support a model in which uptake of fatty acids activates PPARδ-dependent transcription of CPT1A and CPT1A-dependent FAO. Subsequently, increased production of Ac-CoA and the acetylation of β-catenin promote Wnt signaling and cancer stem cell function. Thus, pharmacological inhibition of CPT1A with ETO or knockdown of CPT1A expression may block fatty acids-induced tumor promoting effects in colon cancer (Supplementary Fig. S6D).

## Discussion

Several large prospective epidemiological studies have provided strong evidence supporting the role of obesity in promoting colon cancer initiation and progression^5,32,33^. However, the molecular mechanism underlying how adipose tissue and adipocytes support tumor growth and progression remains largely unknown. We have shown previously that abundant adipocytes are found in direct contact with colon cancer cells and uptake of fatty acids released by adipocytes promotes cancer cell survive by upregulating mitochondrial FAO^8^. Results from this study demonstrate that the presence of adipocytes or fatty acids stimulates the expression of CPT1A by activating PPARδ-dependent transcription. Silencing CPT1A expression in colon cancer cells blocks the cell survival advantage provided by adipocytes as a result of decreased fatty acid degradation via FAO. In addition, CPT1A downregulation induces differentiation of tumor organoids grown in 3D and attenuates the effect of fatty acids on promoting the expression of cancer stem cell-associated genes. Importantly, CPT1A expression is required for adipocytes to promote tumor growth and initiation in vivo.

The cancer stem cells, also known as tumor-initiating cells, have been implicated in tumor initiation, recurrence, and metastasis^34–36^. Emerging evidence suggests that Wnt/β-catenin signaling is required for the maintenance of normal and cancer stem cells^37^. Despite having activating mutations in the Wnt pathway, colon cancer cells with the highest levels of β-catenin signaling display cancer stem cell properties^23^. Consequently, the expression levels of β-catenin target genes have been used as a readout for the stemness of cancer stem cells^38^. Recent studies have begun to establish the role of FAO in regulating normal and cancer stem cell function. For example, it has been shown that CPT1A-dependent FAO is required for the maintenance of tumor initiation cells in hepatocellular carcinoma as well as normal hematopoietic and neural stem cells^39–41^. Moreover, deletion of Cpt1a in mouse intestine epithelium decreases the number and function of normal intestinal stem cells^42^. Our study identifies CPT1A upregulation as a key metabolic alteration that cancer cells adapt to promote β-catenin acetylation and activation in an adipocyte-enriched TME. Since uptake of fatty acids induces the acetylation of proteins other than β-catenin, additional studies are needed to determine the functional contribution of these acetylation events. It is of particular interest to further investigate the epigenetic alterations associated with histone acetylation changes induced by fatty acids.

In addition, it has been shown recently that CPT1 complex may support cell proliferation independent of its ability to control FAO^43^. Although we show that inhibition of FAO is coupled with decreased cell survival and β-catenin activation in CPT1A knockdown cells, it is possible that CPT1A mediates the tumor promoting effects of adipocytes using a FAO-independent mechanism. Moreover, a recent study reported that circulating fatty acid-binding protein released by adipose tissue (A-FABP or FABP4) promotes breast cancer stemness by activating the IL-6/STAT3/ALDH1 pathway^44^. The importance of FABP4 in facilitating the transport of fatty acids from adipocytes to cancer cells has also been shown in ovarian and colon cancers^7,8^. Results from our study here provide strong evidence supporting a role of CPT1A in controlling the production of signaling metabolites to mediate the communication between adipocytes and colon cancer cells. Future studies are needed to further determine the role of FABP4 in regulating cancer stemness in colon cancer.

In summary, results from our study identify CPT1A as a key regulator that connects adipocyte-mediated regulation of cellular metabolism to Wnt signaling in colon cancer cells. Given the preferential upregulation of CPT1A by adipocytes, our findings indicate that inhibition of CPT1A may provide an effective approach to block the tumor promoting effects of adipocytes in colon cancer.

## Supplementary information

Supplemental Figure Legends

Supplemental Figure S1

Supplemental Figure S2

Supplemental Figure S3

Supplemental Figure S4

Supplemental Figure S5

Supplemental Figure S6
