# Supplementary material for: Upregulation of CPT1A is essential for the tumor-promoting effect of adipocytes in colon cancer
Source: Cell Death Dis. 2020 Sep 10;11(9):736. doi: 10.1038/s41419-020-02936-6 (PMC7484798; doi:10.1038/s41419-020-02936-6)
Supplement: Supplementary file 1 — Supplemental Figure Legends [file 41419_2020_2936_MOESM1_ESM.docx]

**Supplemental figure legends**

**Figure S1. The expression of CPT1A in colorectal cancer patient specimens.** The expression of CPT1A protein was detected in colon cancer patient tissues using IHC staining. Three different stage IV colon adenocarcinoma patients, including BH1576, BH1591 and BH1710, were analyzed (A-C). The black- and blue-boxed regions shown in panels A-C were enlarged and presented in panels A’-C’ and A”-C”, respectively. Scale bar, 100 μm.

**Figure S2. Knockdown of CPT1A inhibits fatty acid oxidation.** Control and CPT1A knockdown PT130 were subjected to Seahorse FAO analysis using OA as the metabolic substrate. The relative levels of OCR associated with basal FAO, FAO capacity and FAO reserve were quantified. Data represent the mean ± SD (n=3, * p < 0.01 and ¶ p < 0.0001).

**Figure S3. Downregulation of CPT1A increases glucose metabolism in colon cancer cells.** (A) Representative OCR measurements obtained from the Mito stress test performed in control (sh-Control) and CPT1A knockdown (sh-CPT1A-C6 and sh-CPT1A-C7) PT130 cells using the Seahorse XF96 Extracellular Flux analyzer. Oligomycin, FCCP and rotenone were added at the indicated points. (B) Experiments as shown in (A) were quantified and the relative levels of OCR associated with basal and maximal respiration and ATP production were calculated. Data represent the mean ± SD (n=3, # p < 0.05, § p < 0.001 and ¶ p < 0.0001). (C) Representative OCR measurements obtained from the Mito stress test performed in control (sh-Control) and CPT1A knockdown (sh-CPT1A-C6 and sh-CPT1A-C7) SW480 cells using the Seahorse XF96 Extracellular Flux analyzer. (D) Experiments as shown in (C) were quantified and the relative levels of OCR associated with basal and maximal respiration and ATP production were calculated. Data represent the mean ± SD (n=3, ¶ p < 0.0001). (E) Representative ECAR measurements obtained from the glycolysis stress test performed in control and CPT1A knockdown PT130 cells using the Seahorse XF96 Extracellular Flux analyzer. Glucose, oligomycin and 2-deoxyglucose (2-DG) were added at the indicated points. (F) Experiments as shown in (E) were quantified and ECAR associated with glycolysis, glycolytic capacity and glycolytic reserve was calculated based on the measurements obtained upon the addition of individual compounds. Data represent the mean ± SD (n=3, # p < 0.05 and * p < 0.01). (G) Control and CPT1A knockdown SW480 cells were subjected to the glycolysis stress test using the Seahorse XF96 Extracellular Flux analyzer. (H) Experiments as shown in (E) were quantified and ECAR associated with glycolysis, glycolytic capacity and glycolytic reserve was calculated based on the measurements obtained upon the addition of individual compounds. Data represent the mean ± SD (n=3, # p < 0.05, * p < 0.01 and § p < 0.001).

**Figure S4. Silencing CPT1A did not affect the proliferation of colon cancer cells under regular growth condition.** Equal numbers of control and CPT1A knockdown PT130 (A) and SW480 (B) cells were cultured in regular growth media for 1-4 days. The number of cells were counted at each time point. The rate of cell growth was similar in CPT1A knockdown cells compared to control cells.

**Figure S5. Inhibition of FAO reduces colony formation and cell proliferation in tumor organoids.** (A) Tumor organoids derived from Apc/Kas double mutant mice were pretreated with ETO (20 μM) for 2 days and subsequently viable cells were seeded as single cells in 3D Matrigel. Representative images of control and Cpt1a knockdown tumor organoids are shown after 6 days in culture. Scale bar, 100 μm. (B) The number of colonies formed and the percentage of organoids showed branching phenotype were quantified (total 1,000 cells were seeded per group). Data represent the mean ± SD (n=3, # p < 0.05). (C-D) Knockdown of Cpt1a inhibits cell proliferation without decreasing cell viability in 3D tumor organoids. Same numbers of control and Cpt1a knockdown Apc/Kras tumor cells were seeded in Matrigel and allowed to grow into organoids for 3-10 days. At each time point, the number of viable cells were counted using Trypan blue exclusion method. (C) The rate of cell proliferation was decreased in Cpt1a knockdown tumor organoids. Data represents mean ± SD (n=3, # p < 0.05 and * p < 0.01). (D) The percentage of viable cells was not altered in Cpt1a knockdown tumor organoids.

**Figure S6. Downregulation of Cpt1a decreases β-catenin activation and protein acetylation.** (A) Protein lysates from control and Cpt1a knockdown Apc/Kras mouse tumor organoids were analyzed for the levels of active-β-catenin as well as Ac-α-tubulin, H3K9Ac and H3K27Ac using Western blotting. Total β-catenin, α-tubulin, histone H3 and β-actin were used as loading controls. (B) Control and CPT1A knockdown PT130 cells were treated with BSA or OA (100 μM) for 24 h and cell lysates were analyzed for the levels of active-β-catenin as well as Ac-α-tubulin and H3K9Ac using Western blotting. Total β-catenin, α-tubulin, histone H3 and β-actin were used as loading controls. (C) Control and CPT1A knockdown PT130 cells were cultured under control condition or treated with octanoate (100 μM) for 24 h and cell lysates were analyzed for the levels of active-β-catenin as well as Ac-α-tubulin, H3K9Ac and H3K27Ac using Western blotting. (D) Results from this study support a model in which uptake of fatty acids activates PPARδ-dependent transcription of CPT1A. Subsequently, CPT1A-mediated FAO increases the production of Ac-CoA and the acetylation of β-catenin which promotes Wnt signaling and cancer stem cell function. Pharmacological inhibition of CPT1A with etomoxir or knockdown of CPT1A expression blocks fatty acids-induced tumor promoting effects in colon cancer.

**Supplemental Table S1. Oligonucleotides used for RT-PCR analysis**

|  | **Name** | **Forward (5’-3’)** | **Reverse (5’-3’)** |
| --- | --- | --- | --- |
| Human genes | *ACTB* | CATGTACGTTGCTATCCAGGC | CTCCTTAATGTCACGCACGAT |
|  | *LGR5* | TCAGTCAGCTGCTCCCGAAT | CGTTTCCCGCAAGACGTAAC |
|  | *MYC* | CGTCTCCACACATCAGAGCACAA | TCTTGGCAGCAGGATAGTCCTT |
|  | *PPARGC1A* | TGAAGACGGATTGCCCTCATT | GCTGGTGCCAGTAAGAGCTT |
| Mouse genes | *Actb* | GGCTGTATTCCCCTCCATCG | CCAGTTGGTAACAATGCCATGT |
|  | *Axin2* | TGACTCTCCTTCCAGATCCCA | TGCCCACACTAGGCTGACA |
|  | *Krt20* | AGTTTTCACCGAAGTCTGAGTTC | GTAGCTCATTACGGCTTTGGAG |
|  | *Lgr5* | TGCCCATCACACTGTCACTGT | CACCCTGAGCAGCATCCTG |
|  | *Muc2* | ATGCCCACCTCCTCAAAGAC | GTAGTTTCCGTTGGAACAGTGAA |
|  | *Myc* | TGAGCCCCTAGTGCTGCAT | AGCCCGACTCCGACCTCTT |
|  | *Ppargc1a* | TATGGAGTGACATAGAGTGTGCT | CCACTTCAATCCACCCAGAAAG |
|  | *Tcf7* | AGCTTTCTCCACTCTACGAACA | AATCCAGAGAGATCGGGGGTC |
