# Supplementary figures and images for: Upregulation of CPT1A is essential for the tumor-promoting effect of adipocytes in colon cancer
Source: Cell Death Dis. 2020 Sep 10;11(9):736. doi: 10.1038/s41419-020-02936-6 (PMC7484798; doi:10.1038/s41419-020-02936-6)

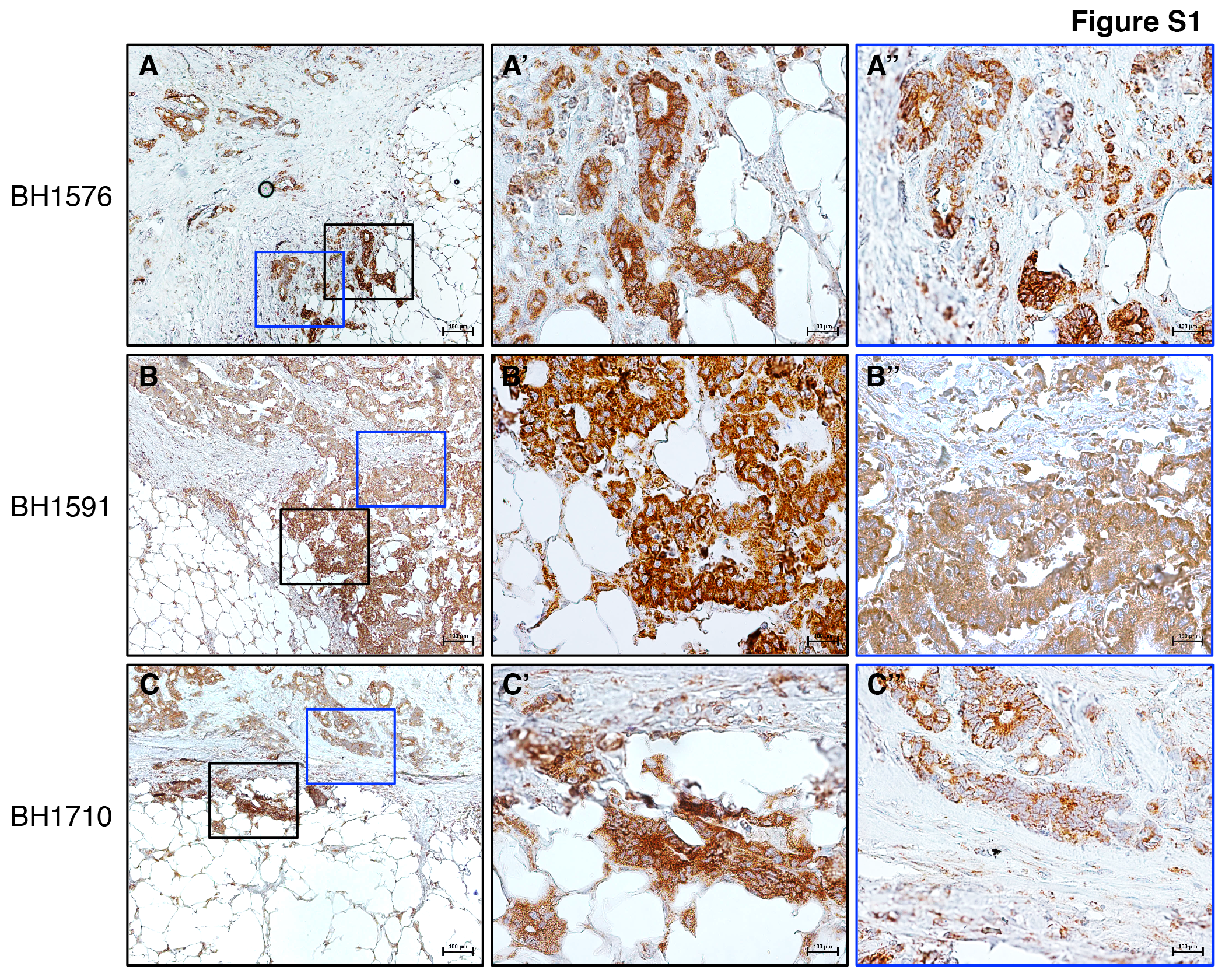

Supplement: Supplementary file 2 — Supplemental Figure S1 [file 41419_2020_2936_MOESM2_ESM.tif]

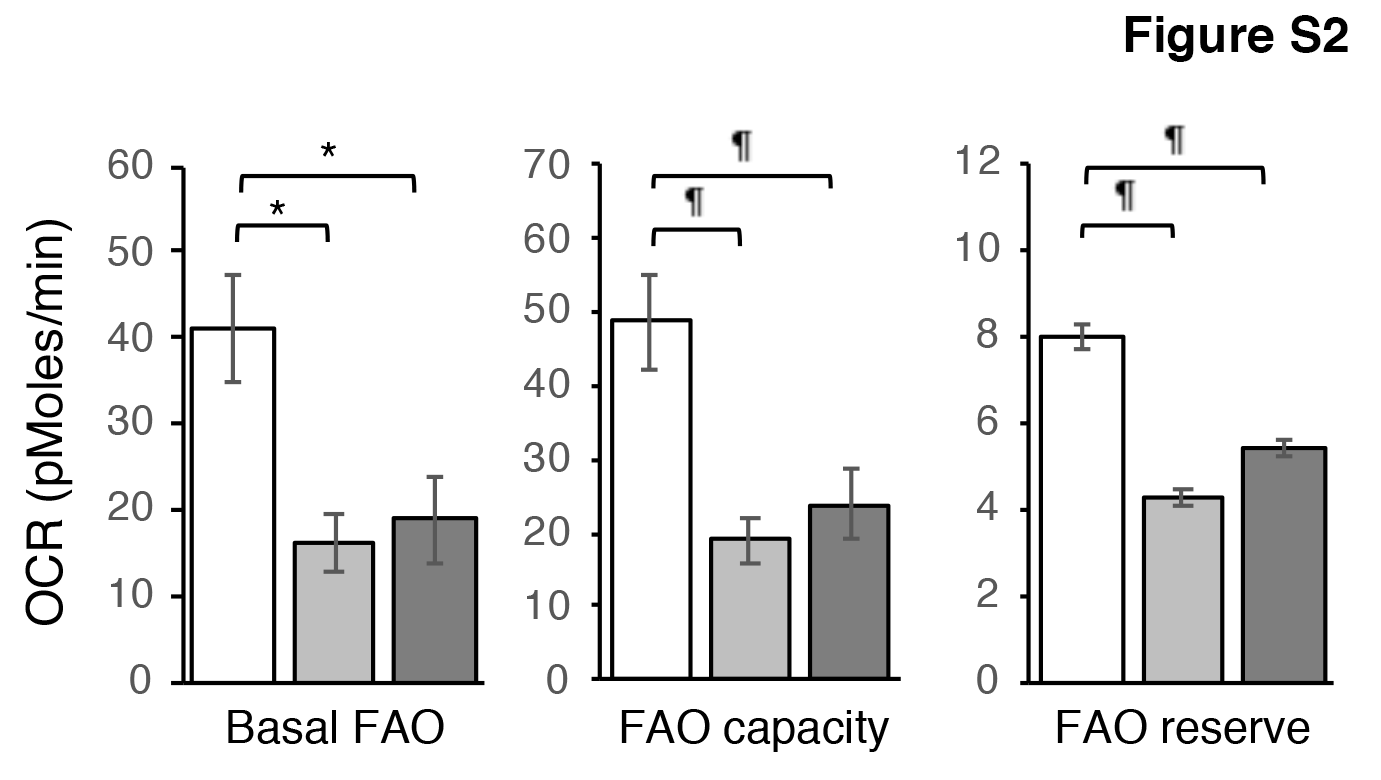

Supplement: Supplementary file 3 — Supplemental Figure S2 [file 41419_2020_2936_MOESM3_ESM.tif]

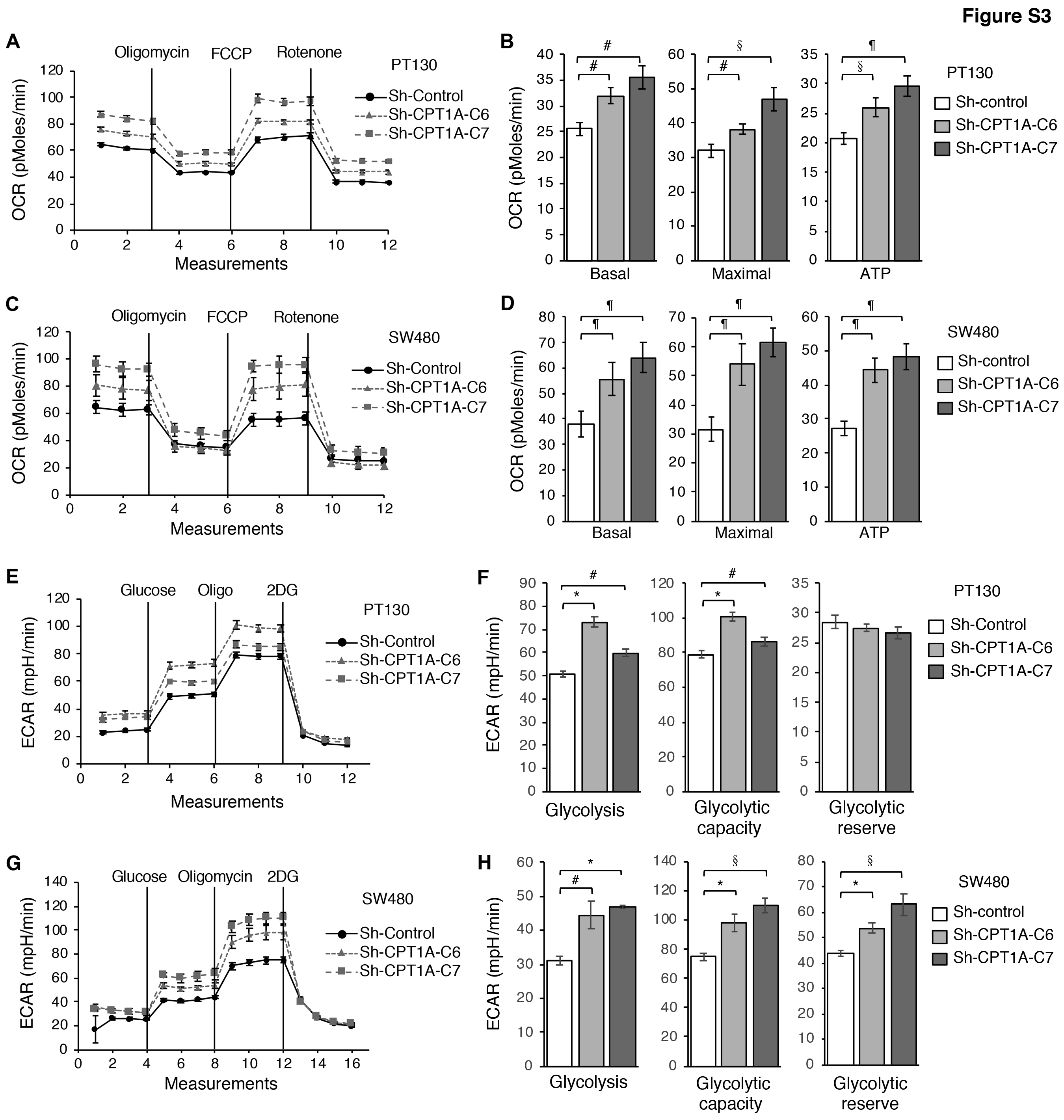

Supplement: Supplementary file 4 — Supplemental Figure S3 [file 41419_2020_2936_MOESM4_ESM.tif]

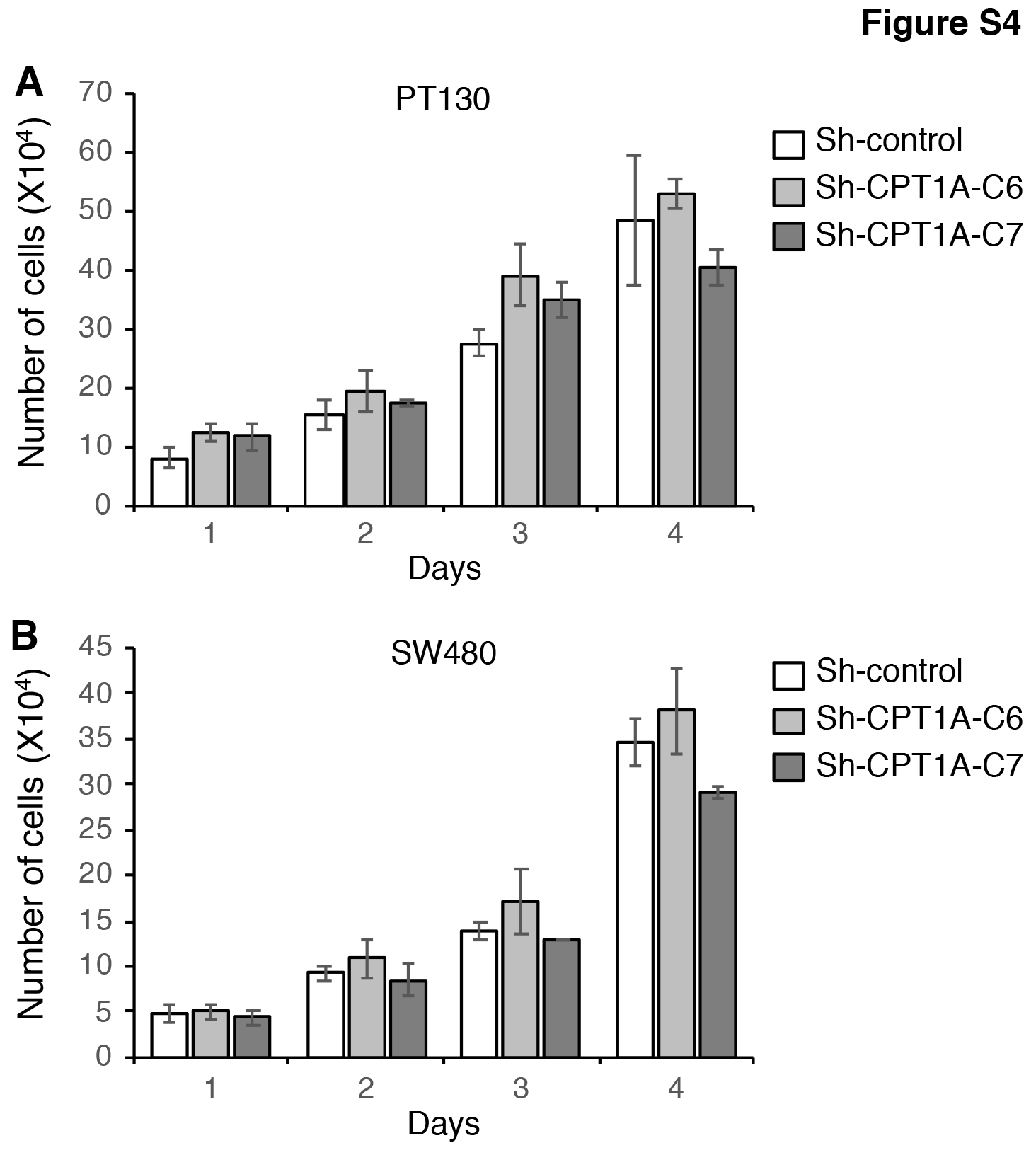

Supplement: Supplementary file 5 — Supplemental Figure S4 [file 41419_2020_2936_MOESM5_ESM.tif]

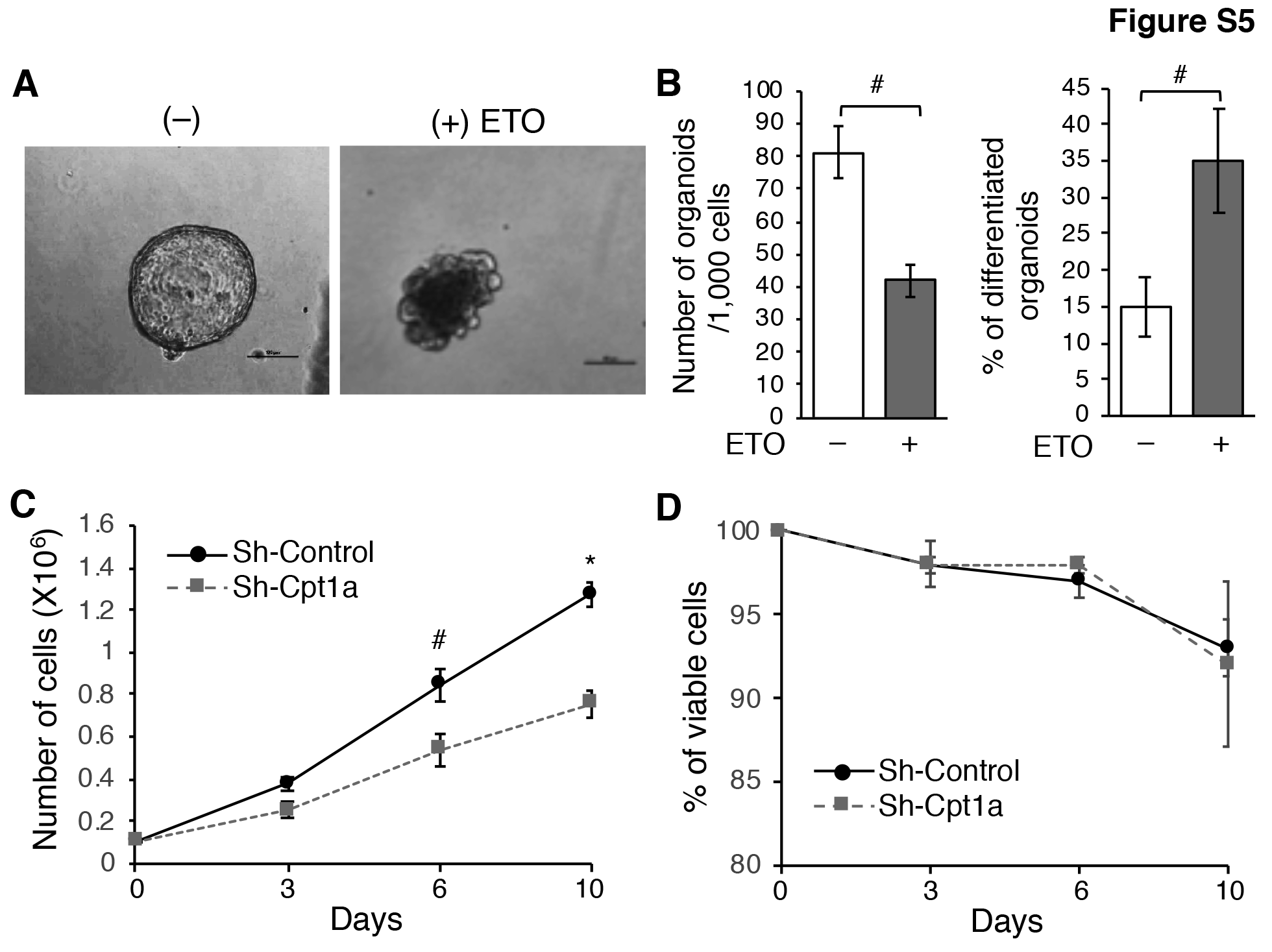

Supplement: Supplementary file 6 — Supplemental Figure S5 [file 41419_2020_2936_MOESM6_ESM.tif]

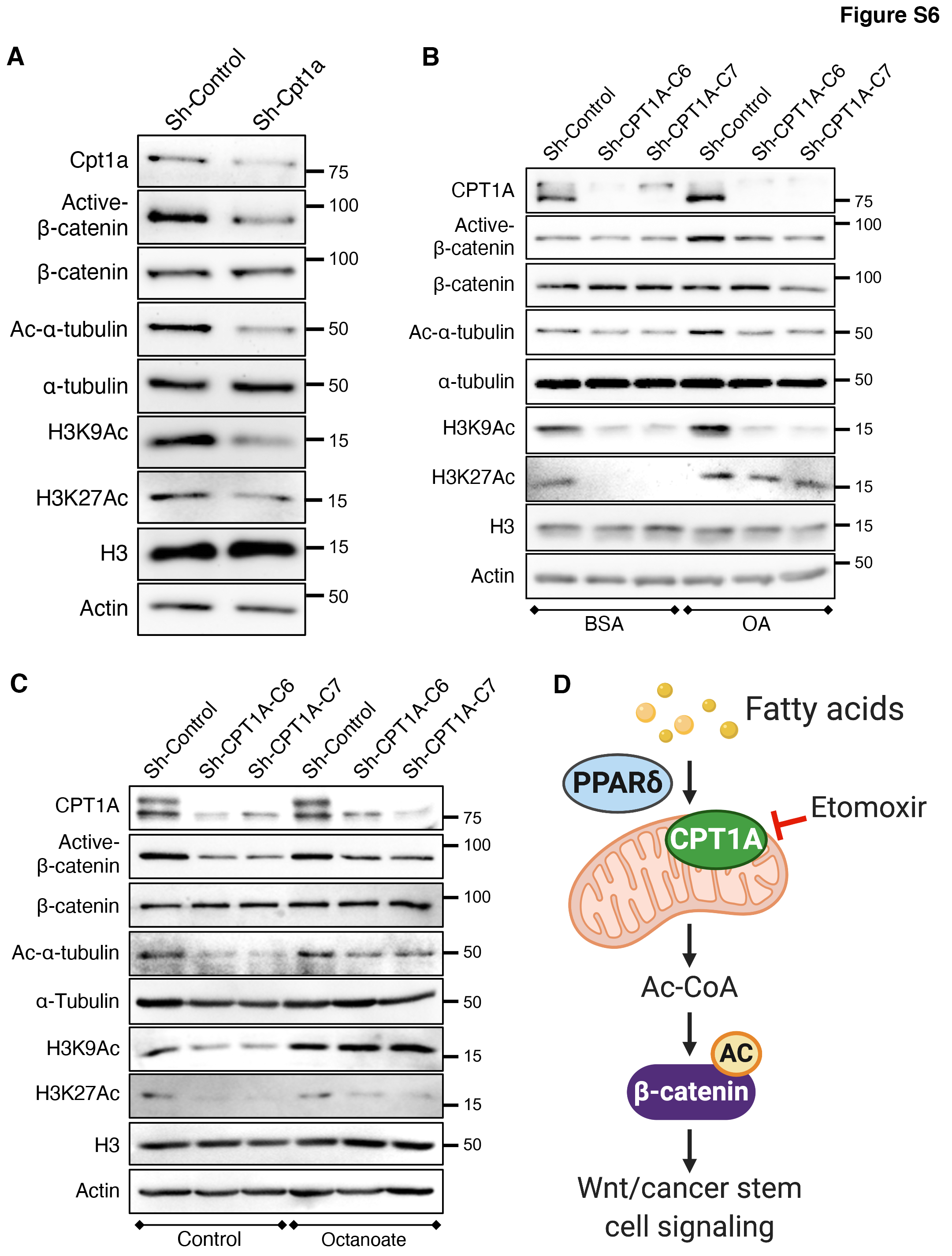

Supplement: Supplementary file 7 — Supplemental Figure S6 [file 41419_2020_2936_MOESM7_ESM.tif]
